# Pediatric Non-Alcoholic Fatty Liver Disease (NAFLD): Trends, Mortality, and Socioeconomic Disparities in the U.S., 1998–2020

**DOI:** 10.3390/children12010071

**Published:** 2025-01-08

**Authors:** Paul Wasuwanich, Joshua M. So, Mustafa Sadek, Chaowapong Jarasvaraparn, Songyos Rajborirug, Ruben E. Quiros-Tejeira, Wikrom Karnsakul

**Affiliations:** 1 Department of Internal Medicine, Naples Comprehensive Health, Naples, FL 34102, USA; 2 Department of Internal Medicine, University of Florida College of Medicine, Gainesville, FL 32610, USA; 3 Division of Gastroenterology, Hepatology and Nutrition, Department of Pediatrics, Riley Hospital for Children, Indiana University School of Medicine, Indianapolis, IN 46202, USA; 4 Division of Pediatric Gastroenterology, Hepatology, and Nutrition, Department of Pediatrics, Johns Hopkins University School of Medicine, 550 N. Broadway 10th Floor Suite 1003, Baltimore, MD 21205, USA; 5 Division of Gastroenterology, Hepatology and Nutrition, Department of Pediatrics, University of Nebraska Medical Center, Omaha, NE 68198, USA

**Keywords:** fatty liver disease, risk factors, diabetes, obesity, coagulopathy, mortality

## Abstract

Background/Objectives: We aim to describe the changing inpatient epidemiology of NAFLD in the U.S. and identify major risk factors associated with mortality in the disease among hospitalized pediatric patients. Methods: Hospitalization data from the 1998–2020 National Inpatient Sample were utilized. ICD-9 and ICD-10 codes were used to identify pediatric patients (age less than 18 years old) with NAFLD, and risk factors for mortality were analyzed by logistic regression. Results: We identified 68,869 pediatric hospitalizations involving NAFLD. Among those, 970 (1.4%) died during hospitalization. Hospitalization rates have been rapidly increasing from 1998 to 2020 (incidence rate ratio (IRR): 1.07; 95% CI: 1.06–1.07; *p* < 0.001). There was a significant difference in mortality based on the type of hospital (rural, non-teaching urban, or teaching urban) in pediatric patients with NAFLD (*p* < 0.05). Coagulopathy was significantly associated with increased odds of mortality, while age ≥ 12 years, diabetes and obesity were associated with decreased odds of mortality (*p* < 0.05). Sex, race/ethnicity, hepatitis B, hepatitis C, HIV, and IV drug use were not significantly associated with mortality. Conclusions: Our study has shown ever increasing hospitalization rates for NAFLD in pediatric populations and well as significant risk factors associated with mortality. Further studies should be performed as more data on this patient population are collected.

## 1. Introduction

What is known:

- Non-alcoholic fatty liver disease (NAFLD) is linked to obesity and type 2 diabetes.
- The prevalence of NAFLD has been increasing in children due to rising obesity rates.
- NAFLD hospitalizations are more common among certain demographics, particularly Hispanic people.

What is new:

- Mortality rates in pediatric NAFLD decreased from 1998 to 2020, despite increasing hospitalization rates.
- Risk factors associated with decreased mortality include older age (≥12 years), diabetes, and obesity, while coagulopathies significantly increase the risk of mortality.
- Non-Hispanic Black children have the highest mortality rate from NAFLD, although they have lower hospitalization rates than Hispanic and non-Hispanic White children.

Non-alcoholic fatty liver disease (NAFLD) is a disease spectrum that involves the accumulation of fat in liver cells (steatosis) in individuals who do not consume significant amounts of alcohol. The spectrum ranges from simple hepatic steatosis to nonalcoholic steatohepatitis (NASH), advanced fibrosis, and cirrhosis [1]. The pathophysiology of NAFLD involves a complex interplay of metabolic, genetic, and environmental factors. The initial step in NAFLD is the accumulation of triglycerides within hepatocytes, primarily due to increased free fatty acid influx from adipose tissue, enhanced de novo lipogenesis, and reduced fatty acid oxidation and export. Insulin resistance plays a central role in this process by promoting lipolysis in adipose tissue, increasing the delivery of free fatty acids to the liver, and enhancing hepatic lipogenesis [2,3].

Progression from simple steatosis to NASH involves multiple parallel hits, including oxidative stress, mitochondrial dysfunction, endoplasmic reticulum (ER) stress, and inflammation. Oxidative stress results from the overproduction of reactive oxygen species (ROS) during fatty acid oxidation, leading to lipid peroxidation, mitochondrial damage, and further hepatocyte injury [2,4]. ER stress, triggered by the accumulation of misfolded proteins, activates the unfolded protein response, which can lead to inflammation and apoptosis if unresolved [5].

Inflammation in NAFLD is driven by the release of pro-inflammatory cytokines and chemokines from injured hepatocytes and activated Kupffer cells. This inflammatory milieu promotes the activation of hepatic stellate cells, leading to fibrosis and, in advanced cases, cirrhosis [3,6]. Several risk factors contribute to the development and progression of NAFLD, including metabolic, genetic, and lifestyle-related variables. Obesity, both in terms of general adiposity (high BMI) and visceral fat accumulation, is a primary risk factor, with studies showing that the prevalence of NAFLD in individuals with severe obesity may exceed 90% [7]. Type 2 diabetes mellitus is another significant risk factor, with up to 69% of individuals with type 2 diabetes mellitus affected by NAFLD [7]. Dyslipidemia, characterized by elevated serum triglycerides and low levels of high-density lipoprotein (HDL) cholesterol, is commonly observed in NAFLD patients, exacerbating hepatic fat accumulation [8]. Hypertension, frequently seen in individuals with NAFLD, may contribute to the progression of the disease [9]. Additionally, genetic factors, such as polymorphisms in genes like PNPLA3 and TM6SF2, have been associated with an increased risk of NAFLD [10]. The prevalence of NAFLD also increases with age, with older individuals being at higher risk, and male gender is identified as a risk factor, as men tend to have a higher incidence of the disease compared to women [7]. Ethnic differences in NAFLD prevalence are notable, with Hispanic individuals showing a higher prevalence than non-Hispanic whites, while non-Hispanic blacks have a lower prevalence [7]. Components of metabolic syndrome, including central obesity, dyslipidemia, hypertension, and insulin resistance, strongly correlate with NAFLD [9]. A sedentary lifestyle and poor physical condition are modifiable risk factors that contribute to the development of the disease, as physical inactivity exacerbates obesity and insulin resistance [11]. Dietary factors, particularly high intake of fructose and other processed sugars, significantly contribute to the development of NAFLD by promoting insulin resistance and hepatic fat accumulation [12].

The spectrum of NAFLD includes a range of liver conditions, from simple steatosis to more severe forms such as NASH, fibrosis, and cirrhosis. Simple steatosis is the initial stage of NAFLD, characterized by the accumulation of fat in the liver without significant inflammation or hepatocellular injury. This stage is generally considered benign, with a low risk of progression to more severe liver disease [7]. NASH is a more advanced form of NAFLD, marked by hepatic steatosis accompanied by inflammation and hepatocyte injury, or ballooning. NASH can occur with or without fibrosis and is a critical stage, as it can progress to cirrhosis and liver failure [7]. Fibrosis develops in NASH when ongoing inflammation and hepatocyte injury activate hepatic stellate cells, leading to extracellular matrix deposition. Fibrosis is staged from F0 (no fibrosis) to F4 (cirrhosis), with its presence and severity being key predictors of liver-related outcomes and overall mortality [13]. Cirrhosis represents the end-stage of chronic liver disease, characterized by extensive fibrosis and the formation of regenerative nodules. Patients with cirrhosis are at increased risk of liver-related complications [14], including liver failure, portal hypertension, and hepatocellular carcinoma (HCC). Advanced fibrosis and cirrhosis can lead to significant complications such as liver decompensation, HCC, and the need for liver transplantation. NAFLD is increasingly becoming a leading cause of liver transplantation and HCC globally [13]. The gold standard diagnostic tool for NAFLD is liver biopsy [14]; however, in practice, it is typically diagnosed from imaging studies or unexplained elevated transaminase as a diagnosis of exclusion in the context of clinical obesity [15].

In the general population, as of 2020, up to 30% of the United States (U.S.) population is estimated to be affected with NAFLD [16]. Due to the rise of obesity in children, the prevalence of NAFLD in children has been increasing as well. From 2017 to 2020, around 19.7% of children in the U.S. are estimated to have had obesity [17], and 4.5–9.6% NAFLD [18,19,20].

Although there have been multiple studies on the pathophysiology, clinical features, and management of NAFLD, there remains a significant gap in understanding its morbidity and mortality, particularly in the pediatric population. The purpose of this study is to investigate the epidemiology of NAFLD during hospitalization in U.S. pediatric patients under 18 years old and examine trends from 1998 to 2020. Additionally, this study aims to explore the demographic, clinical, and hospital-related factors associated with mortality in hospitalized pediatric NAFLD patients. By analyzing data from a large, national cohort, this study seeks to provide valuable insights into the key risk factors that impact outcomes for this population, ultimately informing strategies to improve clinical management and reduce mortality rates associated with NAFLD in children. This research will help guide future interventions, refine diagnostic criteria, and shape policies aimed at improving care for children with NAFLD.

## 2. Materials and Methods

### 2.1. Study Population

After receiving approval from the Johns Hopkins University School of Medicine’s institutional review board, we utilized data from the National Inpatient Sample (NIS) provided by the Healthcare Cost and Utilization Project (HCUP). The NIS is a stratified sample that includes approximately 20% of all hospitals in the United States participating in HCUP, representing a broad and diverse array of hospitals, both academic and non-academic. The NIS captures around 20% of more than 35 million discharges annually, providing a comprehensive dataset for examining healthcare trends and outcomes across various patient populations. For our study, we analyzed a 23-year interval, spanning from 1998 to 2020, to assess trends in pediatric NAFLD hospitalizations and related mortality risk factors.

### 2.2. Data Extraction

Data for pediatric NAFLD cases were extracted using diagnostic codes from the International Classification of Diseases, Ninth Revision (ICD-9) and Tenth Revision (ICD-10). NAFLD-associated hospitalizations were identified using the ICD-9 code 571.8 and ICD-10 codes K75.81 and K76.0. Pediatric patients were defined as those aged 18 years or younger, and no exclusions were made based on race/ethnicity or sex.

A comprehensive set of demographic variables was included in the analysis, including age, sex, race/ethnicity, and geographic region/division of the hospital. The type of hospital—whether rural, urban non-teaching, or urban teaching—was also noted. In addition to these demographic factors, clinical data such as length of hospital stay (in days) and all-cause mortality rates were extracted to assess the severity and outcomes of hospitalizations.

Given the common association between obesity and diabetes mellitus (including both type 1 and type 2) and NAFLD, data on these two conditions were specifically included in the analysis. Furthermore, additional factors potentially influencing liver pathologies, such as coagulopathies, HIV infection, hepatitis B virus (HBV) infection, and hepatitis C virus (HCV) infection, were also collected for examination. These variables were considered to provide a comprehensive view of the factors influencing morbidity and mortality in pediatric patients with NAFLD.

### 2.3. Statistical Analysis

Hospitalization rates of NAFLD were analyzed for trends between 1998 and 2020 using Poisson regression and reported as an incidence rate ratio (IRR) for overall annual change. Hospitalization rate calculations incorporated discharge weights provided by HCUP to provide the true number of hospitalizations, compensating for the 20% sampling of the database. Changes in discharge weights prior to 2012 due to database redesign were accounted for in the hospitalization rates. The denominator used in the calculation of hospitalization rates was the total United States population residing within the country on July 1st of the relevant year. These data were extracted from the database of the United States Census. NAFLD cases were identified based on ICD codes appearing at any location in the diagnosis list, including both primary and secondary diagnoses. Additionally, we explored changes in racial/ethnic distributions and sex distributions of NAFLD over time.

Case-fatality rates were calculated as the percentage of deaths that resulted from all hospitalizations for a given disease. The non-normal data were summarized using the median and interquartile range (IQR) and compared using the Mann–Whitney U test. Frequencies were compared using the chi-squared test.

Risk factors for morbidity and mortality among NAFLD hospitalizations were analyzed by logistic regression in both univariable (crude) and multivariable (adjusted) models. Variables with *p*-values below 0.2 on univariable analysis were then selected for multivariable analysis. The dichotomous outcomes were alive at discharge versus death. Results were reported by odds ratio (OR) with 95% confidence intervals (CI). Odds ratios are derived from exponentiated logistic regression coefficients. For a continuous variable, the odds ratio represented the odds for each unit increase of that variable.

All results reported are weighted, using discharge weights provided by HCUP to present the true number of hospitalizations. Results for a category that contained greater than zero but 10 or fewer hospitalizations were displayed as ≤10 due to the data use privacy policy of HCUP. Missing data were assumed to be missing at random.

Statistical significance was defined as *p* < 0.05. Statistical calculations were performed using the R program (R version 4.4.1).

## 3. Results

**Figure 1:**
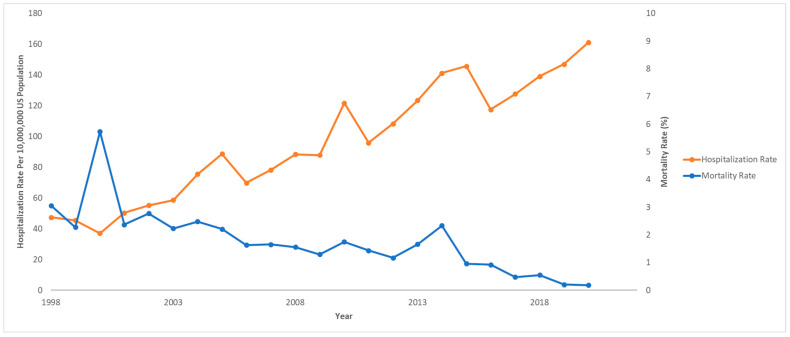
Hospitalization rates and mortality rates over time for pediatric non-alcoholic fatty liver disease (NAFLD). National Inpatient Sample, Healthcare Cost and Utilization Project (HCUP), 1998–2020.

**Figure 2:**
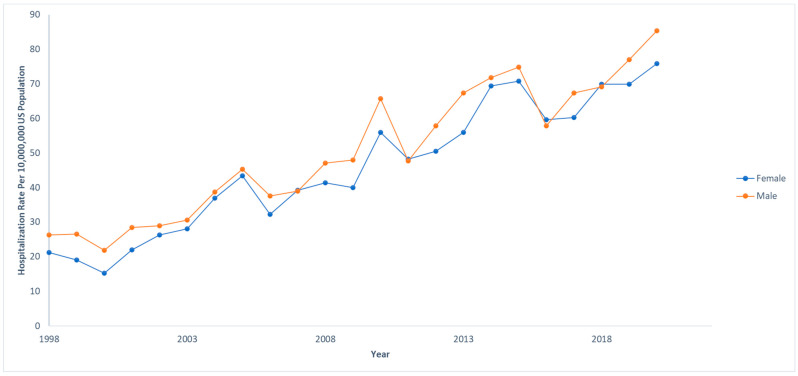
Hospitalization rates over time for pediatric non-alcoholic fatty liver disease (NAFLD) by sex. Each sex was compared to the total US population at that given year. National Inpatient Sample, Healthcare Cost and Utilization Project (HCUP), 1998–2020.

**Figure 3:**
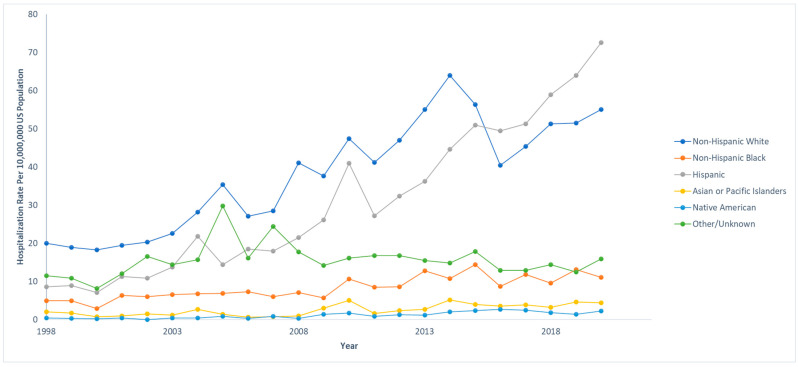
Hospitalization rates over time for pediatric non-alcoholic fatty liver disease (NAFLD) by race/ethnicity. Each race/ethnicity was compared to the total US population in that given year. National Inpatient Sample, Healthcare Cost and Utilization Project (HCUP), 1998–2020.

Out of 833,177,022 total hospitalizations reported in the U.S. from 1998 to 2020, we identified 68,869 pediatric hospitalizations involving NAFLD. Among those who were hospitalized with NAFLD, 970 (1.4%) died. The hospitalization rate per 10,000,000 people in the U.S. has steadily increased from 47.4 to 161.3 from 1998 to 2020 (IRR: 1.07; 95% CI: 1.06–1.07; *p* < 0.001). However, the mortality rate has decreased from 3.1% to 0.2% in the same time span. The hospitalization and mortality rates are shown in Figure 1. Hospitalization rates by stratified by sex are displayed in Figure 2, and hospitalization rates stratified by race/ethnicity are shown in Figure 3. Although not statistically significant, the race/ethnicity with the greatest mortality rate are non-Hispanic Black subjects at 2.1%. The hospitalization rate of non-Hispanic Black patients was found to be 11.1%, which is lower than Hispanic and non-Hispanic White populations.

Among our study cohort, 36,101 (52.4%) were male, and the median age was 13 (IQR: 6–16) years. Most of the cohort were non-Hispanic White patients (n = 36,101; 39.3%) and were hospitalized in the Southern region of the U.S. (n = 25,142; 36.5%). Most cases of pediatric NAFLD were hospitalized in urban teaching hospitals (n = 55,514; 80.6%), and most deaths were in these hospitals as well (n = 828; 85.4%). Upon further examination of the data, younger age was associated with mortality; the median age of patients who died was 4 [IQR: 0–12] years compared to 13 [IQR: 7–16] years among those who survived (*p* < 0.001). Hospitalization at an urban teaching center (*p* = 0.037) was also significantly associated with increased mortality. Sex, race/ethnicity, and region of the hospital were not significantly associated with mortality rates (*p* > 0.05) (Table 1).

A logistic regression analysis of demographic, social, and clinical factors on the mortality of pediatric NAFLD patients was conducted (Table 2). In the univariable analysis, we found that age ≥ 12 years (OR = 0.42; 95% CI = 0.30–0.59; *p* < 0.001), diabetes mellitus (OR = 0.34; 95% CI = 0.13–0.92; *p* = 0.033), and obesity (OR = 0.15; 95% CI = 0.07–0.34; *p* < 0.001) were significantly associated with decreased odds of mortality. However, underlying coagulopathy (OR = 4.52; 95% CI = 3.10–6.57; *p* < 0.001) was found to be significant associated with increased odds in mortality. Sex, race/ethnicity, hepatitis B, hepatitis C, HIV, and intravenous drug use were not significantly associated with mortality.

In the multivariable analysis for variables with *p*-values under 0.2 from the univariable analysis, age ≥ 12 years (OR = 0.41; 95% CI = 0.30–0.57; *p* < 0.001), diabetes mellitus (OR = 0.30; 95% CI = 0.11–0.82; *p* = 0.019), and obesity (OR = 0.18; 95% CI = 0.08–0.37; *p* < 0.001) were still significantly associated with decreased odds of mortality. Underlying coagulopathy (OR = 4.42; 95% CI = 3.10–6.29; *p* < 0.001) was also significantly associated with increased odds in mortality. Race/ethnicity, hepatitis B, HIV, and intravenous drug use were not significantly associated with mortality in the multivariable analysis (Table 2).

## 4. Discussion

This study represents the largest analysis of the epidemiology and mortality risk factors associated with hospitalized pediatric NAFLD patients in the United States. To our knowledge, it is currently the only study that investigates mortality risk factors beyond the commonly recognized factors of obesity, diabetes, age, sex, and race/ethnicity in the U.S. pediatric population. Our findings contribute valuable insights into the broader spectrum of factors influencing outcomes in pediatric NAFLD [21]. In 2022, Dybbro et al. conducted a study investigating hospitalizations of pediatric patients with NAFLD [21]. While the study provided valuable insights, it had several limitations. The scope of clinical factors explored was narrow, and the analysis was confined to a limited timeframe. Additionally, the study did not examine the morbidity or mortality associated with NAFLD [21], which are essential components for understanding the full impact of the disease in pediatric populations. These gaps highlight the need for further, more comprehensive research in this field.

Rates of hospitalization for pediatric patients with NAFLD have been steadily increasing over time [21]. This trend is largely attributed to the rising prevalence of obesity and diabetes in children, which are major risk factors for the development of NAFLD. As both conditions become more common, the number of pediatric NAFLD cases continues to rise, contributing to the increase in hospitalizations within this patient population, highlighting the growing burden of the disease [14,17,21,22].

However, our study found an interesting trend in that mortality rates for children with NAFLD were decreasing over time from 1998 to 2020. This observation is particularly significant, as it suggests improvements in the outcomes for this patient population. One of the main factors likely contributing to this decrease is the evolution of early detection methods and interventions for both diabetes and obesity.

Children with NAFLD who also have diabetes and obesity often benefit from closer medical surveillance and targeted interventions, which can reduce the risk of mortality. These comorbidities prompt healthcare providers to implement lifestyle modifications, medications, or both, which not only manage the primary conditions but also mitigate the progression of liver disease and associated complications. These early interventions may have beneficial effects on liver health, reducing hepatic fat accumulation and inflammation. This combination of early detection, intensive care, and potentially liver-protective treatments could explain the decreased odds of mortality observed in these children.

Early detection allowed healthcare providers to manage NAFLD and its associated risk factors more effectively, leading to better long-term outcomes and a reduction in mortality of NAFLD [23,24,25].

Moreover, we speculated that lean NAFLD could also be a contributing factor to the observed decrease in mortality among children with obesity related NAFLD. Research has suggested that while lean individuals with NAFLD (defined by a BMI ≤ 25.0 kg/m^2^) do not typically exhibit the same components of metabolic syndrome as those with obesity-related NAFLD, they may still be at an elevated risk of mortality [26]. A study by Feldman et al. highlighted that these lean individuals with NAFLD are more likely to have poor liver-related outcomes despite their lower BMI. This suggests that factors beyond obesity, such as genetic predispositions or hepatic inflammation, could influence outcomes in this subset of patients.

Interestingly, a meta-analysis by Liu et al. in 2019 showed that both adult and pediatric NAFLD patients are at an elevated risk of all-cause mortality, independent of factors such as age, weight loss, diabetes, smoking, or hypertension [25], emphasizing the need for further research to understand the complex mechanisms underlying NAFLD-related mortality.

We have found that hospitalization rates were the greatest in the Hispanic population, which is consistent with similar studies that analyzed the demographics in this population [27]. The race/ethnicity with the greatest mortality rate was non-Hispanic Black patients at 2.1%, although this was not found to be statistically significant. Interestingly, the hospitalization rate of non-Hispanic Black patients was 11.1%, which was lower than that of Hispanics and non-Hispanic White patients. According to the National Health Statistics Report by the Centers for Disease Control and Prevention (CDC), non-Hispanic Black patients have the second highest obesity prevalence at 24.8%, right below Hispanic patients (26.2%). This discrepancy may be explained by socioeconomic inequalities in income and education within the non-Hispanic Black population, as well as a culture of mistrust in the U.S. medical system which could result in non-Hispanic Black families being less likely than other groups to seek medical care for their children [28,29]. This disproportionate effect may also partly result from limited access to healthcare, which impedes early detection and intervention. Socioeconomic challenges often restrict routine medical visits, causing children to miss early screenings for NAFLD. This allows the disease to progress silently, advancing to more severe forms like NASH or cirrhosis and leading to poorer outcomes in this population. Addressing healthcare access disparities is essential to improving early detection and reducing the burden of NAFLD.

Younger age (<12 years) was significantly associated with increased mortality in pediatric NAFLD patients. This finding may be explained by the fact that genetic and hereditary lipid disorders are often linked to the earlier development of NAFLD and a worse prognosis. Children with these conditions may experience more rapid progression of liver damage, contributing to higher mortality rates. Additionally, genetic factors could play a role in how the liver responds to fat accumulation, further exacerbating the disease’s severity in younger [30,31]. Our study also found that urban teaching hospitals had the highest number of pediatric NAFLD patients between 1998 and 2020, as well as disproportionately higher mortality rates compared to other types of hospitals. This observation may be due to the fact that smaller, non-teaching hospitals tend to transfer more complicated or critically ill patients to academic medical centers, where they receive specialized care. As a result, urban teaching hospitals may care for more severe cases, potentially influencing the mortality outcomes in this setting.

Our study highlights that coagulopathies significantly increase mortality in hospitalized pediatric patient with NAFLD, independent of traditional risk factors like diabetes and obesity, commonly seen in adults. Even in the absence of cirrhosis, which is uncommon in the pediatric population, early advanced liver disease in NAFLD can lead to disruptions in the production of both pro-coagulation and anti-coagulation factors, increasing the risk for both thrombosis and hemorrhage which can contribute to mortality [32]. Unlike simple steatosis or early-stage NASH, where liver function remains largely intact, more severe stages are likely to introduce complications related to hemostasis, elevating the risk of mortality. While we believe the relationship between NAFLD and coagulopathies to be closely related, it is possible that some of these patients may have died from their underlying coagulopathy without complications from NAFLD. Fibrosis stage, identified as a critical predictor of liver-related outcomes by the American Association of Clinical Endocrinologists and the American Association for the Study of Liver Diseases, underscores the connection between advanced liver damage and coagulopathy [13].

Surveillance for these complications is vital in patients with advanced fibrosis or cirrhosis to mitigate risks [33]. Research also indicates that liver-related deaths in advanced NAFLD, including fatalities linked to coagulopathy complications like variceal hemorrhage and hepatic decompensation, are significant contributors to mortality [34]. Hence, diligent management and monitoring of coagulopathy in pediatric NAFLD patients are essential to prevent fatal outcomes.

More studies should be done to understand etiology of coagulopathy and its effects on pediatric NAFLD morbidity and mortality.

HIV was initially found to be significantly associated with mortality in the pediatric NAFLD cohort. However, when adjusted for other variables, this association was no longer present, suggesting that HIV may not be an independent risk factor for mortality in this population. Between 1998 and 2020, only 14 pediatric NAFLD patients with HIV died, and the limited number of cases could likely explain the non-significant finding after adjusting for other factors.

There were some limitations to our study that must be considered when interpreting the results. One of the primary concerns is information bias. The National Inpatient Sample (NIS) database, while extensive and invaluable for large scale, population-based research, lacks standardized diagnostic criteria for NAFLD. The absence of a consistent approach to diagnosing NAFLD across different healthcare providers could lead to variability in how the disease is identified and coded, which introduces the possibility of information bias. Specifically, the lack of standardized diagnostic protocols may result in inconsistent or inaccurate diagnoses, particularly when clinicians rely on clinical judgment or subjective criteria. While we assume that any miscoding or differences in coding practices would be randomly distributed across the study sample and thus would not significantly affect the mortality outcome, this assumption may not always be valid. Misclassification could potentially skew the data, especially if certain cases are underreported or over-diagnosed, and this limitation should be kept in mind when interpreting the findings.

Another limitation of our study stems from the temporal variation in NAFLD diagnosis over the past several years. As awareness of metabolic diseases, including NAFLD, has increased, more cases, particularly those with milder symptoms or as a comorbidity, may be diagnosed. This increasing recognition of NAFLD may reflect a positive shift in diagnostic practices, as healthcare providers become more attuned to its identification and management. However, this growing awareness could have introduced a potential bias in our study, as the rising number of diagnoses could contribute to the observed upward trend in NAFLD prevalence, particularly among individuals with less severe manifestations of the disease. Conversely, earlier recognition and intervention could lead to a reduction in mortality rates, as individuals with milder forms of NAFLD are diagnosed sooner and potentially treated before their condition worsens. The interplay between increasing awareness and improving outcomes could explain the rising trend in NAFLD cases while simultaneously bringing down the mortality rate, making it difficult to draw definitive conclusions about the true impact of NAFLD on mortality over time.

The pathophysiology of NAFLD also presents a limitation in our study. NAFLD is a chronic, slowly progressive disease, and the full impact on mortality may not be immediately observable, especially in cases that progress over many years. This slow progression of the disease poses challenges in determining its direct role in hospital stay mortality, particularly for patients who may have other significant comorbidities. The limited data on pediatric NAFLD further complicates this issue, as the effects of NAFLD on pediatric populations are not as well-documented in the available database. Additionally, pediatric cases of NAFLD could coexist with other risk factors, such as genetic metabolic disease, which may significantly influence mortality outcomes. Unfortunately, the NIS database does not provide sufficient information to fully assess the interactions between NAFLD and these other conditions. As a result, our study may not capture the full scope of mortality related to pediatric NAFLD, and the true burden of this disease in younger populations could be underestimated.

Furthermore, the reliance on ICD-9 and ICD-10 codes for mortality data is another limitation of our study. These codes do not distinguish whether a death is directly caused by NAFLD or by another comorbid condition. The presence of multiple diseases in patients with NAFLD complicates the attribution of death solely to NAFLD. This lack of specificity makes it challenging to definitively attribute in-hospital mortality to NAFLD alone, as patients often have multiple comorbidities, such as cardiovascular disease, diabetes, or chronic kidney disease, which could have contributed to their death. As a result, we cannot definitively state that NAFLD was the primary cause of death for each patient in our study. This limitation underscores the complexity of determining the direct relationship between NAFLD and mortality in real world clinical settings.

Lastly, although recent guidelines have shifted from the term NAFLD to metabolic associated fatty liver disease (MAFLD) to better reflect its underlying pathophysiology, the ICD-9 and ICD-10 coding systems have not yet adopted this change. Consequently, we continued to use the term NAFLD in our study to maintain consistency with the coding system in place during the study period. This inconsistency in terminology could lead to some confusion, as the updated terminology is not yet reflected in clinical practice. However, despite this issue, our study still provides valuable insights into the trends of NAFLD diagnosis and its associated mortality outcomes, although the evolving terminology should be kept in mind when interpreting the findings.

## 5. Conclusions

NAFLD is an increasingly prevalent and complex condition in the pediatric population. Hospitalization rates for pediatric NAFLD have been rising steadily, reflecting the growing burden of this disease. However, an interesting trend observed in our study was a decrease in mortality rates over time, suggesting potential improvements in the management and early detection of the condition. Our study identified several significant risk factors associated with mortality in pediatric NAFLD patients, including age, diabetes, obesity, HIV, and coagulopathies. These findings provide valuable insights into the key determinants of adverse outcomes in this population. Understanding these risk factors is essential for developing targeted interventions and improving the management of pediatric NAFLD. Moving forward, our findings should serve as a foundation for further research aimed at refining current guidelines and improving clinical outcomes for pediatric patients with NAFLD.

## Figures and Tables

**Table 1:** Demographic and geographic characteristics of nationwide pediatric non-alcoholic fatty liver disease (NAFLD) hospitalizations. National Inpatient Sample, Healthcare Cost and Utilization Project (HCUP), 1998–2020.

Characteristics	Total	NAFLD Alive	NAFLD Died	*p*-Value
**Number of Hospitalizations, N**	**68,869**	**67,899**	**970**	
Age, Year, Median (IQR)	13 (6–16)	13 (7–16)	4 (0–12)	**<0.001**
Sex				0.193
Male, N (%)	36,101 (52.4)	35,683 (52.6)	418 (43.1)	
Female, N (%)	32,768 (47.6)	32,216 (47.4)	552 (56.9)	
Race/Ethnicity				
Non-Hispanic White, N (%)	27,080 (39.3)	26,689 (39.3)	391 (40.3)	0.126
Non-Hispanic Black, N (%)	5918 (8.6)	5796 (8.5)	122 (12.6)	
Hispanic, N (%)	22,329 (32.4)	22,070 (32.5)	259 (26.7)	
Asian or Pacific Islander, N (%)	1793 (2.6)	1754 (2.6)	39 (4.0)	
Native American, N (%)	811 (1.2)	807 (1.2)	≤10	
Other/Unknown, N (%)	10,938 (15.9)	10,783 (15.9)	155 (16.0)	
Region of Hospital				0.839
Northeast, N (%)	10,023 (14.6)	9871 (14.5)	152 (15.7)	
Midwest, N (%)	11,785 (17.1)	11,640 (17.1)	145 (14.9)	
South, N (%)	25,142 (36.5)	24,805 (36.5)	337 (34.7)	
West, N (%)	19,968 (29.0)	19,680 (29.0)	288 (29.7)	
Type of Hospital				**0.037**
Rural, N (%)	2349 (3.4)	2334 (3.4)	15 (1.5)	
Urban Non-Teaching, N (%)	8746 (12.7)	8671 (12.8)	75 (7.7)	
Urban Teaching, N (%)	55,514 (80.6)	54,686 (80.5)	828 (85.4)	

**Table 2:** Selective logistic regression multivariable analysis of demographic, social, and clinical factors with a p-value of <0.20 on univariable analysis of pediatric non-alcoholic fatty liver disease (NAFLD) hospitalizations on mortality, 1998–2020.

Risk Factors		Crude Odds Ratio (95% CI)	Crude *p*-Value	Adjusted Odds Ratio (95% CI)	Adjusted *p*-Value
Demographics					
Age	≥12 (vs. <12)	0.25 (0.19–0.35)	**<0.001**	0.41 (0.30–0.57)	**<0.001**
Sex	Female (vs. Male)	0.83 (0.63–1.10)	0.194	0.92 (0.69–1.23)	0.577
Race/Ethnicity	White (vs. Other)	1.03 (0.77–1.39)	0.839	-	-
Metabolic Disorders					
Diabetes	Yes (vs. No)	0.12 (0.05–0.33)	**<0.001**	0.30 (0.11–0.82)	**0.019**
Obesity	Yes (vs. No)	0.10 (0.05–0.21)	**<0.001**	0.18 (0.08–0.37)	**<0.001**
Co-Infections					
Hepatitis B	Yes (vs. No)	5.97 (0.78–46.15)	0.087	4.84 (0.70–33.47)	0.110
Hepatitis C	Yes (vs. No)	3.03 (0.41–22.55)	0.278	-	-
HIV Positive	Yes (vs. No)	4.03 (1.24–13.11)	**0.021**	2.85 (0.85–9.52)	0.089
Social Factors					
Intravenous Drug Use	Yes (vs. No)	2.73 (0.66–11.28)	0.165	2.41 (0.48–12.15)	0.288
Other					
Coagulopathy	Yes (vs. No)	6.12 (4.36–8.59)	**<0.001**	4.42 (3.10–6.29)	**<0.001**

## Data Availability

The data presented in this study are available on request from the corresponding author due to privacy restrictions from the Healthcare Cost and Utilization Project (HCUP).

## Abbreviations and Acronyms

United States (U.S.); non-alcoholic fatty livers disease (NAFLD); non-alcoholic steatohepatitis (NASH); Healthcare Cost and Utilization Project (HCUP); confidence interval (CI); odds ratio (OR).
